# Interleukin-32: its role in asthma and potential as a therapeutic agent

**DOI:** 10.1186/s12931-018-0832-x

**Published:** 2018-06-25

**Authors:** Tong Xin, Mo Chen, Liwei Duan, Yanling Xu, Peng Gao

**Affiliations:** 1 Department of Respiratory Medicine, the Second Hospital of Jilin University, Changchun, Jilin, China; 2 Department of Gastrointestinal medicine, the Second Hospital of Jilin University, Changchun, Jilin, China; 3 Department of Geriatrics and General Medicine, the Second Hospital of Jilin University, Changchun, Jilin, China

**Keywords:** Interleukin-32, Asthma, Therapeutic agent, Anti-inflammation, Pro-inflammation

## Abstract

Interleukin (IL)-32, also named natural killer cell transcript 4 (NK4), has increasingly been described as an immunoregulator that controls cell differentiation and cell death and is involved in the stimulation of anti−/pro-inflammatory cytokines. Abnormal presence of IL-32 has been repeatedly noticed during the pathogenesis of allergic, infectious, cancerous, and inflammatory diseases. Of particular note was the observation of the anti-inflammatory property of IL-32 in a murine ovalbumin model of allergic asthma. Compared to wild-type mice, IL-32γ transgenic mice show decreased levels of inflammatory cells, recruited eosinophils, and lymphocytes in bronchoalveolar lavage fluid in a mouse model of acute asthma. To date, the molecular mechanism underlying the role of IL-32 in asthma remains to be elucidated. This review aims to summarize recent advances in the pathophysiology of asthma and describe the links to IL-32. The possibilities of using IL-32 as an airway inflammation biomarker and an asthma therapeutic agent are also evaluated.

## Background

Interleukin (IL) -32, a cytokine that was identified in 1992 and originally called natural killer cell transcript 4 (NK4) [1–3], is involved in the pathogenesis of various disorders including allergic, infectious, cancerous, and inflammatory diseases. Kim et al. (2005) found that IL-32 can induce the production of some inflammatory cytokines (i.e., IL-8 and tumor necrosis factor alpha [TNF-α]) [4]. Since then, the potential biological functions of IL-32 have been widely investigated. High-dose IL-2 can induce the mRNA expression of IL-32 in peripheral blood mononuclear cells (PBMCs), especially NK and mitogen-stimulated T cells [3, 5]. Other cytokines, such as interferon gamma (IFN-γ), IL-18, TNF-α, and Th1 cell cytokines can also induce the production of IL-32 [5, 6]. The immunoregulatory functions of IL-32 have increasingly been mentioned in the recent literature, with studies showing that IL-32 influences cell differentiation [7–9] and cell death [10, 11] and is involved in the stimulation of anti−/pro-inflammatory cytokines [12–14]. IL-32 may exert its function through both extracellular and intracellular pathways. While the specific surface IL-32 receptor remains to be identified, it is widely accepted that IL-32 can bind to proteinase 3 and integrins (e.g., αVβ3 and αVβ6) [15, 16]. In the presence of functional αVβ3, recombinant IL (rIL)-32γ induces endothelial cell tube formation in vitro [17]. The intracellular receptors for IL-32 have not yet been discovered either. In AGS cell lines co-cultured with Helicobacter pylori, IL-32 was not detected by ELISA in supernatants, while high levels of IL-32 was found in both cytosol and nuclear [18]. These results are in line with the studies that demonstrated intracellular IL-32 expression and leakage from apoptotic cells [10, 19, 20].

**Fig. 1:**
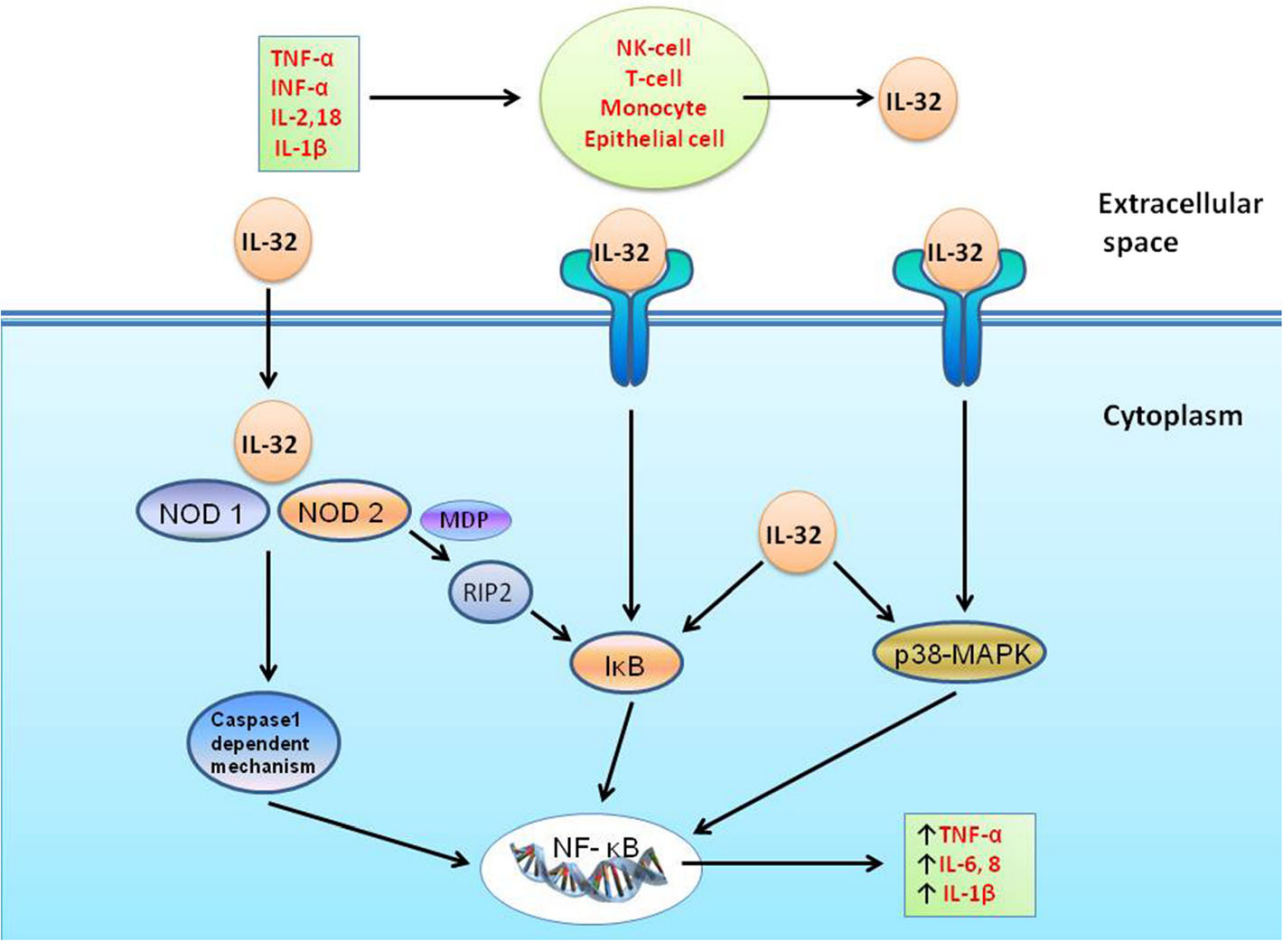
Production of IL-32 and its downstream signaling network. IL-32 is produced by a variety of cells (e.g., NK cells, T cells, monocytes, and epithelial cells), and its production can be stimulated by different cytokines (e.g., TNF-α, INF-α, IL-2, IL-18, and IL-1β). IL-32 synergizes with NOD1 and NOD2 and subsequently induces the production of IL-6 and IL-1β via a caspase-1–dependent signaling pathway. IL-32 can also activate NF-κB signaling through the IκB pathway (via NOD2-MDP or directly acting on IκB) and/or the p38-MAPK pathway

The location of the human IL-32 gene is in chromosome 16p13.3. Interestingly, the IL-32 gene has not been found in rodents. The lack of a mouse model has highly limited the possibility of investigating IL-32 function in vivo [21]. Appropriate approaches to study IL-32 would be helpful, including but not limited to 3D cell culture, ex vivo human lung, organoid-like models and lung on a chip. IL-32 can promote the production of IL-8 through the nuclear factor-kappa B (NF-κB) and the p38 mitogen-activated protein kinase (MAPK) pathways (Fig. 1) [4]. Intracellular nucleotide-binding oligomerization domain (NOD) proteins 1 and 2 can also synergize with IL-32 and induce the production of IL-6 and IL-1β via a caspase 1-dependent signaling pathway (Fig. 1) [22]. To date, a total of nine IL-32 isoforms (α, β, γ, δ, ε, ζ, η, θ, ι) have been identified [4, 10, 23]. Recent studies indicated that these IL-32 isoforms have different biological activities and properties compared to the other isoforms [23–27]. For example, IL-32γ shows an effective antiviral property against viruses like human immunodeficiency virus, herpes simplex virus 2, influenza A virus, and vesicular stomatitis virus [28–32]. IL-32β can upregulate IL-10 production through the protein kinase C (PKC) δ pathway [27]. However, IL-32δ can suppress the binding of IL-32β to PKCδ and thereby decrease the IL-32β–induced IL-10 production. In other words, the biological activities of IL-32 are regulated by its own isoforms [27].

IL-32 is involved in the pathogenesis of a number of chronic inflammatory diseases and allergic diseases including but not limited to rheumatoid arthritis (RA), chronic obstructive pulmonary disease (COPD), COPD exacerbation, inflammatory bowel disease (IBD), chronic rhinosinusitis and asthma (Table 1) [1, 6, 21, 33, 34]. In the synovial tissues of RA patients, IL-32 is highly expressed and positively correlated with disease activity [35]. In addition to synovial macrophages, synovial fibroblasts can also produce TNF-α, which is a potent inducer of IL-32. Interestingly, the mRNA transcription levels of IL-1β and TNF-α were stabilized when IL-32 was overexpressed in these cells. In addition, a decreased level of IL-32 was detected in the synovial tissue biopsies when RA patients started anti-TNF-α therapy [12]. Thus, the interactions between TNF-α and IL-32 should be seriously investigated in RA patients. Moreover, in the patients with seasonal allergic rhinitis, dsRNA challenge increased IL-32 expression when compared to saline challenge at the height of the pollen season [36]. In bronchial epithelial cells, IL-32 was induced by dsRNA via the NF-κB signaling pathway. Also, IL-32 is involved in the pathogenesis of airway inflammation [37]. In allergic asthma, Rhinovirus infection could induce the expressions of the inflammation-related genes and IL-32 [38].

**Table 1:** The presence of IL-32 in blood/tissue fluid of patients with inflammatory diseases

Disease	Assay	IL-32 level in blood/tissue fluid (pg/ml)	Comments
COPD/Asthma	ELISA (BioLegend, USA)	Serum: healthy controls, 4.6 ± 1.0; COPD, 26.8 ± 2.6; asthma, 6.1 ± 1.2. Broncho-alveolar lavage: healthy controls, 4.2 ± 1.1; COPD, 22.5 ± 2.5; asthma, 6.3 ± 1.1. Induced sputum: healthy controls, 3.6 ± 0.7; COPD, 19.7 ± 1.7; asthma, 5.8 ± 1.2.	Patients with COPD had increased levels of IL-32 when compared to asthma patients and healthy controls [62].
SLE	ELISA (R&D Systems, USA)	Plasma: healthy controls, 94.4 (40.2–233.7); SLE, 34.7 (15.5–140.5).	SLE patients had lower levels of IL-32 than healthy controls [63]. This decreased level may be associated with drug treatment and the chemotherapy-related bone marrow cytotoxicity.
HF	ELISA (Hermes Criterion Biotechnology, Canada)	Serum: healthy controls, 111 ± 59; HF, 237 ± 92.	Patients with HF had higher levels of IL-32 than healthy controls [64].
BD	ELISA (R&D Systems, USA)	Serum: healthy controls, 0.1 (0.1–14.7); BD, 0.4 (0.1–736.2).	BD patients had higher levels of IL-32 than healthy controls [65].
*Helicobacter pylori* GI/GC	ELISA (BioLegend, USA)	Gastritis tissue: healthy controls, 208 ± 133; GI, 643 ± 492; GC, 1651 ± 488.	Patients with *Helicobacter pylori*-induced GI/GC had higher levels of IL-32 than healthy controls. GC patients had higher levels of IL-32 than GI patients [18].
RA/OA	ELISA (Biosource International, USA)	Synovial fluid: RA, 107.5 ± 50.9; OA, 14.4 ± 5.9.	Patients with RA has higher levels of IL-32 than those with OA [66].
MM	ELISA (R&D Systems, USA)	Plasma: healthy controls, 112 ± 45; MM, 1103 ± 345.	Compared to healthy controls, MM patients had higher levels of IL-32 [67].

*SLE* Systemic Lupus Erythematosus, *HF* Heart Failure, *GI* Gastric Inflammation, *GC* Gastric Cancer, *BD* Behçet’s Disease, *RA* Rheumatoid Arthritis, *OA* Osteoarthritis, *MM* Multiple Myeloma

Data are expressed as mean ± SD or median (IQR)

In recent decades, many studies have been conducted to demonstrate the broad-range functions of IL-32. The abnormal presence of IL-32 has been linked to a variety of diseases/disorders. Here we present recent advances with regard to the role of IL-32 in the pathophysiology of asthma.

### IL-32 and inflammation

IL-32 is produced by a variety of immune cells (e.g., NK cells, T cells, PBMCs, and monocytes) and nonimmune cells (e.g., endothelial cells, fibroblasts, and keratinocytes) [3, 11, 19, 39]. The role of IL-32 in inflammation is pleiotropic, since it is involved in not only promoting pro-inflammatory cytokines but also stimulating anti-inflammatory cytokines [12–14]. IL-32 induces the production of prostaglandin E2, a pro-inflammatory factor, in in vitro systems (i.e., human blood monocytes and mouse macrophages). In naïve mice, knee joint injection of IL-32γ increases knee inflammation and causes joint swelling and cartilage damage via TNF-α–dependent signaling [35]. The level of IL-32 in synovial biopsies from active RA patients is positively correlated with the erythrocyte sedimentation rate, synovial inflammatory status, and synovial levels of pro-inflammation cytokines (i.e., TNF-α, IL-1β, and IL-18) [35]. Similarly, the expression (both mRNA and protein) of IL-32 in nasal mucosa is increased in allergic rhinitis (AR) patients. In AR patients, the nasal mucosa IL-32 production is positively correlated with the production of inflammatory factors (i.e., IL-1β, IL-18, and granulocyte-macrophage colony-stimulating factor [GM-CSF]). The pro-inflammatory function of IL-32 was further confirmed in an AR animal model, in which IL-32 increased the production of IgE and inflammatory cytokines [40]. In lung tissue and plasma samples from COPD patients, IL-32 expression is high and is positively correlated with the severity of airflow obstruction [33, 41]. Further studies have proved that during acute COPD exacerbation, inflammation and oxidative stress can increase the expression of IL-32 in human bronchial epithelial (HBE) cells through the JNK pathway. c-Jun and cAMP response element binding protein play key roles in IFN-γ– and H_2_O_2_–induced IL-32 expression [42]. In addition, the serum IL-32 level is increased in patients with H1N1 influenza infection [43]. Of particular interest is that IL-32 can also inhibit the production of pro-inflammatory factors. For example, IL-32β can stimulate the expression of IL-10, an anti-inflammatory cytokine that can suppress the production of pro-inflammatory cytokines (e.g., IL-12, IL-1β, and TNF-α) [14]. In phorbol myristate acetate (PMA)–treated IL-32θ–expressing THP-1 cells, IL-32θ can interact with PKCδ and induce STAT3 Ser727 phosphorylation, thereby decreasing the transcription of CC chemokine ligand (CCL) 5 [44]. Similarly, in patients with acute myeloid leukemia (AML), the PMA-induced TNF-α expression can be inhibited by IL-32θ [45]. Future studies should focus on the pro- and/or anti-inflammatory functions of each IL-32 isoform.

### IL-32 and monocytes/macrophages

**Fig. 2:**
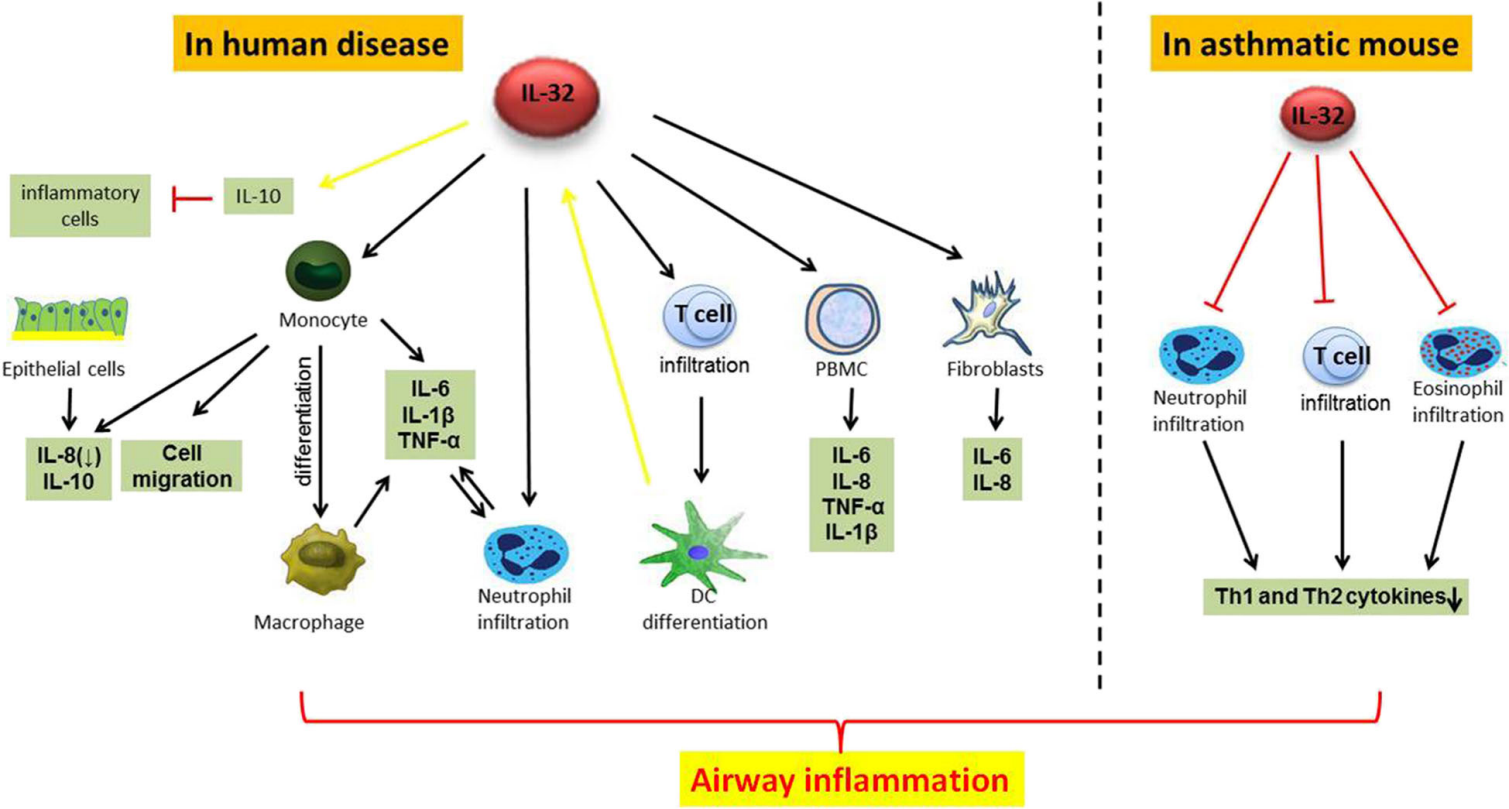
Potential roles of IL-32 in asthma. Positive effects are presented using black arrows, and negative effects are presented using the T-shaped ends. The expression of IL-32 in endothelial cells is indicated using a yellow arrow [61]. Another yellow-arrow is used to indicate that IL-32 can stimulate anti-inflammatory IL-10 expression in dendritic cells [14]. DC, dendritic cell; IL, interleukin

IL-32 is involved in the monocyte-to-macrophage differentiation process (Fig. 2). IL-32 induces the differentiation of human blood monocytes into macrophage-like cells that can phagocytize bacteria. The IL-32–driven monocyte-to-macrophage differentiation is partly dependent on the activity of the caspase-3 proteases. Also, IL-32 can enhance the function of muramyl dipeptide (MDP), a ligand for NOD2 receptor that plays important roles in monocyte-to-macrophage differentiation [7]. However, IL-32θ can inhibit the PMA-induced monocyte-to-macrophage differentiation and inhibit the adhesion capability and morphological change of THP-1 cells. Furthermore, IL-32θ was found to reduce the expression of various macrophage markers, namely, CD11b, CD18, and CD36 [46].

IL-32 stimulates macrophages to produce pro-inflammatory factors (e.g., TNF-α, IL-1β, and IL-6; Fig. 2) via the p38-MAPK and NF-κB pathways [7]. Human IL-32 can promote the production of IL-1β, TNF-α, and macrophage inflammatory protein 2 (MIP-2) in mouse macrophages. Prostaglandin E2 production of human PBMCs can be induced by IL-32 as well [35]. Similarly, the production of IL-1β, IL-6, IL-8, and TNF-α was down-regulated by silencing of IL-32 expression in monocytes [32].

### IL-32 and other cells

In Crohn’s disease (CD) and IBD, IL-32 stimulates the expression of IL-6 and IL-8 in neutrophils, subsequently inducing the productions of pro-inflammatory cytokines in differentiated macrophages and dendritic cells (DCs) and thereby recruiting T cells to the inflamed area. Without the presence of immune suppressor molecules, the concentrated immune cells could induce neutrophil infiltration into the inflamed area. Eventually, the infiltrating neutrophils could release a variety of neutrophil proteinases that cause mucosal tissue damage and augment the inflammation status in CD and IBD patients [8, 21, 47, 48]. IL-32 expression could be detected in nasal mucosa eosinophils. Moreover, in a human eosinophilic leukemia cell line, the expression of IL-32 was highly stimulated by GM-CSF [40]. Furthermore, IL-32γ exhibited synergistic effects [with the presence of iE-DAP (NOD1 ligand) or MDP (NOD2 ligand)] on the induction of allergic inflammation-related IL-1β, TNF-α, and chemokines (CXCL8, CCL3, and CCL4), and on the activation of human eosinophils (via the intracellular caspase 1, ERKs, p38 MAPK, and NF-κB pathways). IL-32γ (with the presence of iE-DAP or MDP) could also increase the cell-surface expressions of adhesion molecule CD18 and ICAM-1 in eosinophils [49].

### IL-32 in animal asthma models

The anti-inflammatory property of IL-32 has been demonstrated in a murine ovalbumin (OVA) model of allergic asthma (Fig. 2) [50]. In this asthma model, in the broncho-alveolar lavage fluid (BALF), the numbers of total inflammatory cells, recruited eosinophils, and lymphocytes were decreased; however, the numbers of macrophages/IL-10–producing CD11b + mononuclear cells were increased in IL-32γ transgenic (TG) mice compared to those in wild-type (WT) mice. There was no difference in BALF neutrophil counts between the two groups. The anti-inflammatory function of IL-32 was confirmed by the histological evaluation of lung tissues after hematoxylin and eosin staining, which showed that inflammatory cell infiltration around the vascular and bronchial areas was decreased in IL-32γ TG mice compared to that in WT mice. Moreover, the histopathologic score of the lung sections indicated a lower degree of peribronchial and perivascular inflammation in IL-32γ TG mice than in WT mice. Also, the expression levels of inflammatory cytokines, including Th2 and Th1 cytokines, were reduced in IL-32γ TG asthma mice.

Similarly, rIL-32γ–treated asthmatic mice had decreased numbers of inflammatory cells (i.e., eosinophils, neutrophils, and lymphocytes) in BALF. Histopathologic examination showed a remarkable suppression of peribronchial and perivascular inflammation in rIL-32γ–treated mice as well [50].

### IL-32 in human asthma

Asthma involves chronic airway inflammation and manifests with airway hyperresponsiveness (AHR) and asthmatic symptoms (e.g., wheezing, breath shortness, and chest tightness) [51]. The airway inflammation is characterized by abnormal responses of T-cells [52], especially CD4^+^ T cells (e.g., Th1, Th2 and Th17) [53].

Asthma is a heterogeneous disease. Basically, asthma can be sorted into four subtypes (neutrophilic, eosinophilic, mixed granulocytic, and paucigranulocytic) according to the presence of inflammatory cells in the induced sputum samples. Thus, each asthma subtype represents a special inflammatory disorder and should be treated with a specific therapy [54–56]. Thus, the underlying molecular mechanisms in the pathophysiology of each asthma subtype need to be thoroughly elucidated for the development of so-called “personalized medicine”. Corticosteroids are currently the first-line therapy for asthma. Eosinophilic asthma, which can be corrected or partially corrected by corticosteroids, is a well-characterized asthma subtype in which the allergen-induced Th2 cytokines (e.g., IL-4, IL-5, IL-9, and IL-13) play key roles [57]. By contrast, neutrophilic asthma, which is resistant to corticosteroid treatment, is driven by the activation of innate immune system proteins such as Toll-like receptors (TLRs) and nucleotide-binding oligomerization domain (NLRP) 3 inflammasome [58, 59]. To date, the underlying molecular mechanisms in the pathophysiology of neutrophilic asthma are much less characterized compared to those of eosinophilic asthma.

IL-32 has increasingly been suggested as a key player in the pathophysiology of asthma. In asthma, the airway presence of IL-32γ is negatively correlated with the forced expiratory volume in 1 s (FEV1) and positively correlated with the annual exacerbation rate [60]. In that study, the authors concluded that the increased IL-32γ level in the induced sputum samples correlated with an increased risk of asthma exacerbation. These results were consistent with those of Meyer et al.’s study, in which the increased sera IL-32 level in asthma patients was accompanied by increased sera levels of pro-inflammatory factors [61]. Because the supernatant of IL-32 siRNA-transfected normal human bronchial epithelial cells increased in vitro angiogenesis of human umbilical vein endothelial cells, IL-32 might be also an inhibitor of airway remodeling in asthma patients [61]. Accordingly, inhibition of IL-32 signal might be a potential therapeutic direction for asthma treatment. Notably, the roles of IL-32 may be different for each subtype of asthma, and these should be further investigated.

From this summary of the recent literature, it is clear that the roles of IL-32 in asthma remain controversial based on the inconsistencies in the results from in vivo or in vitro studies. For example, in the study by Bang et al. (2014), the IL-32 level was decreased [50], but not increased as reported by Meyer et al. (2012) [61], in sputum and serum samples from asthma patients compared to levels in healthy controls. These contradictions might be explained by the nature of asthma airway inflammation (heterogeneity, four subtypes) and the functional differences between IL-32 isoforms. Additional studies should be conducted to investigate the presence of IL-32 isoforms in patients with difference asthma subtypes.

## Conclusion

IL-32 has repeatedly been proposed to be an effective regulator of the systemic inflammatory status both in vivo and in vitro. Targeting of IL-32 signaling might be a potential therapeutic strategy for asthma treatment. While IL-32 has widely been accepted as a pro-inflammatory cytokine, recent studies demonstrate its anti-inflammatory functions as well. These controversial results need to be investigated in upcoming studies.

## Abbreviations

AHR: Airway hyper-responsiveness
AML: Acute myeloid leukemia
AR: Allergic rhinitis
BALF: Bronchoalveolar lavage fluid
BD: Behçet’s disease
CCL: CC chemokine ligand
CD: Crohn’s disease
COPD: Chronic obstructive pulmonary disease
DCs: Dendritic cells
ELISA: Enzyme-linked immunosorbent assay
FEV: Forced expiratory volume
GC: Gastric cancer
GI: Gastric inflammation
GM-CSF: Granulocyte-macrophage colony-stimulating factor
HBE: Human bronchial epithelial
HF: Heart failure
IBD: Inflammatory bowel disease
IFN: Interferon
IL: Interleukin
MAPK: Mitogen-activated protein kinase
MDP: Muramyl dipeptide
MIP: Macrophage inflammatory protein
MM: Multiple myeloma
NF-κB: Nuclear factor-kappa B
NK4: Natural killer cell transcript 4
NLRP: Nucleotide-binding oligomerization domain
NOD: Nucleotide-binding oligomerization domain
OA: Osteoarthritis
OVA: Ovalbumin
PBMCs: Peripheral blood mononuclear cells
PKC: Protein kinase C
PMA: Phorbol myristate acetate
RA: Rheumatoid arthritis
rIL: Recombinant interleukin
SLE: Systemic lupus erythematosus
TG: Transgenic
Th: Helper T cell
TLRs: Toll-like receptors
TNF: Tumor necrosis factor
WT: Wild-type

## References

[1] Hong JT, Son DJ, Lee CK, Yoon DY, Lee DH, Park MH (2017). Interleukin 32, inflammation and cancer. Pharmacol Ther.

[2] Li W, Liu Y, Mukhtar MM, Gong R, Pan Y, Rasool ST (2008). Activation of interleukin-32 pro-inflammatory pathway in response to influenza a virus infection. PLoS One.

[3] Dahl CA, Schall RP, He HL, Cairns JS (1992). Identification of a novel gene expressed in activated natural killer cells and T cells. J Immunol.

[4] Kim SH, Han SY, Azam T, Yoon DY, Dinarello CA (2005). Interleukin-32: a cytokine and inducer of TNFalpha. Immunity.

[5] Panelli MC, Wang E, Phan G, Puhlmann M, Miller L, Ohnmacht GA, et al. Gene-expression profiling of the response of peripheral blood mononuclear cells and melanoma metastases to systemic IL-2 administration. Genome Biol. 2002;3:RESEARCH0035. 10.1186/gb-2002-3-7-research0035 PMC126240 12184809

[6] Soyka MB, Treis A, Eiwegger T, Menz G, Zhang S, Holzmann D (2012). Regulation and expression of IL-32 in chronic rhinosinusitis. Allergy.

[7] Netea MG, Lewis EC, Azam T, Joosten LA, Jaekal J, Bae SY (2008). Interleukin-32 induces the differentiation of monocytes into macrophage-like cells. Proc Natl Acad Sci U S A.

[8] Kim YG, Lee CK, Oh JS, Kim SH, Kim KA, Yoo B (2010). Effect of interleukin-32gamma on differentiation of osteoclasts from CD14+ monocytes. Arthritis Rheum.

[9] Mabilleau G, Sabokbar A (2009). Interleukin-32 promotes osteoclast differentiation but not osteoclast activation. PLoS One.

[10] Goda C, Kanaji T, Kanaji S, Tanaka G, Arima K, Ohno S (2006). Involvement of IL-32 in activation-induced cell death in T cells. Int Immunol.

[11] Meyer N, Zimmermann M, Burgler S, Bassin C, Woehrl S, Moritz K (2010). IL-32 is expressed by human primary keratinocytes and modulates keratinocyte apoptosis in atopic dermatitis. J Allergy Clin Immunol.

[12] Heinhuis B, Koenders MI, van Riel PL, van de Loo FA, Dinarello CA, Netea MG (2011). Tumour necrosis factor alpha-driven IL-32 expression in rheumatoid arthritis synovial tissue amplifies an inflammatory cascade. Ann Rheum Dis.

[13] Heinhuis B, Koenders MI, van de Loo FA, Netea MG, van den Berg WB, Joosten LA (2011). Inflammation-dependent secretion and splicing of IL-32{gamma} in rheumatoid arthritis. Proc Natl Acad Sci U S A.

[14] Kang JW, Choi SC, Cho MC, Kim HJ, Kim JH, Lim JS (2009). A proinflammatory cytokine interleukin-32beta promotes the production of an anti-inflammatory cytokine interleukin-10. Immunology.

[15] Dinarello CA, Kim SH (2006). IL-32, a novel cytokine with a possible role in disease. Ann Rheum Dis.

[16] Heinhuis B, Netea MG, van den Berg WB, Dinarello CA, Joosten LA (2012). Interleukin-32: a predominantly intracellular proinflammatory mediator that controls cell activation and cell death. Cytokine.

[17] Nold-Petry CA, Rudloff I, Baumer Y, Ruvo M, Marasco D, Botti P (2014). IL-32 promotes angiogenesis. J Immunol.

[18] Sakitani K, Hirata Y, Hayakawa Y, Serizawa T, Nakata W, Takahashi R (2012). Role of interleukin-32 in helicobacter pylori-induced gastric inflammation. Infect Immun.

[19] Hasegawa H, Thomas HJ, Schooley K, Born TL (2011). Native IL-32 is released from intestinal epithelial cells via a non-classical secretory pathway as a membrane-associated protein. Cytokine.

[20] Kobayashi H, Lin PC (2009). Molecular characterization of IL-32 in human endothelial cells. Cytokine.

[21] Kim S (2014). Interleukin-32 in inflammatory autoimmune diseases. Immune Netw.

[22] Netea MG, Azam T, Ferwerda G, Girardin SE, Walsh M, Park JS (2005). IL-32 synergizes with nucleotide oligomerization domain (NOD) 1 and NOD2 ligands for IL-1beta and IL-6 production through a caspase 1-dependent mechanism. Proc Natl Acad Sci U S A.

[23] Kang JW, Park YS, Lee DH, Kim MS, Bak Y, Ham SY (2014). Interaction network mapping among IL-32 isoforms. Biochimie.

[24] Chae JI, Shim JH, Lee KS, Cho YS, Lee KS, Yoon DY (2010). Downregulation of immune response by the human cytokines interleukin-32alpha and beta in cell-mediated rejection. Cell Immunol.

[25] Lee DH, Hong JE, Yun HM, Hwang CJ, Park JH, Han SB (2015). Interleukin-32beta ameliorates metabolic disorder and liver damage in mice fed high-fat diet. Obesity (Silver Spring).

[26] Kim SJ, Lee S, Kwak A, Kim E, Jo S, Bae S (2014). Interleukin-32gamma transgenic mice resist LPS-mediated septic shock. J Microbiol Biotechnol.

[27] Kang JW, Park YS, Lee DH, Kim MS, Bak Y, Park SH, et al. Interleukin-32delta interacts with IL-32beta and inhibits IL-32beta-mediated IL-10 production. FEBS Lett. 2013;587:3776–81. 24396867

[28] Li W, Sun W, Liu L, Yang F, Li Y, Chen Y (2010). IL-32: a host proinflammatory factor against influenza viral replication is upregulated by aberrant epigenetic modifications during influenza a virus infection. J Immunol.

[29] Nold MF, Nold-Petry CA, Pott GB, Zepp JA, Saavedra MT, Kim SH (2008). Endogenous IL-32 controls cytokine and HIV-1 production. J Immunol.

[30] Rasool ST, Tang H, Wu J, Li W, Mukhtar MM, Zhang J (2008). Increased level of IL-32 during human immunodeficiency virus infection suppresses HIV replication. Immunol Lett.

[31] Zepp JA, Nold-Petry CA, Dinarello CA, Nold MF (2011). Protection from RNA and DNA viruses by IL-32. J Immunol.

[32] Nold-Petry CA, Nold MF, Zepp JA, Kim SH, Voelkel NF, Dinarello CA (2009). IL-32-dependent effects of IL-1beta on endothelial cell functions. Proc Natl Acad Sci U S A.

[33] Calabrese F, Baraldo S, Bazzan E, Lunardi F, Rea F, Maestrelli P (2008). IL-32, a novel proinflammatory cytokine in chronic obstructive pulmonary disease. Am J Respir Crit Care Med.

[34] Jia TG, Zhao JQ, Liu JH (2014). Serum inflammatory factor and cytokines in AECOPD. Asian Pac J Trop Med.

[35] Joosten LA, Netea MG, Kim SH, Yoon DY, Oppers-Walgreen B, Radstake TR (2006). IL-32, a proinflammatory cytokine in rheumatoid arthritis. Proc Natl Acad Sci U S A.

[36] Brandelius A, Andersson M, Uller L (2014). Topical dsRNA challenges may induce overexpression of airway antiviral cytokines in symptomatic allergic disease. A pilot in vivo study in nasal airways. Respir Med.

[37] Ota K, Kawaguchi M, Fujita J, Kokubu F, Huang SK, Morishima Y (2015). Synthetic double-stranded RNA induces interleukin-32 in bronchial epithelial cells. Exp Lung Res.

[38] Herbert C, Do K, Chiu V, Garthwaite L, Chen Y, Young PM (2017). Allergic environment enhances airway epithelial pro-inflammatory responses to rhinovirus infection. Clin Sci (Lond).

[39] Schenk M, Krutzik SR, Sieling PA, Lee DJ, Teles RM, Ochoa MT (2012). NOD2 triggers an interleukin-32-dependent human dendritic cell program in leprosy. Nat Med.

[40] Jeong HJ, Shin SY, Oh HA, Kim MH, Cho JS, Kim HM (2011). IL-32 up-regulation is associated with inflammatory cytokine production in allergic rhinitis. J Pathol.

[41] Greene CM, Low TB, O'Neill SJ, McElvaney NG (2010). Anti-proline-glycine-proline or antielastin autoantibodies are not evident in chronic inflammatory lung disease. Am J Respir Crit Care Med.

[42] Kudo M, Ogawa E, Kinose D, Haruna A, Takahashi T, Tanabe N (2012). Oxidative stress induced interleukin-32 mRNA expression in human bronchial epithelial cells. Respir Res.

[43] Bae S, Kang D, Hong J, Chung B, Choi J, Jhun H (2012). Characterizing antiviral mechanism of interleukin-32 and a circulating soluble isoform in viral infection. Cytokine.

[44] Bak Y, Kang JW, Kim MS, Park YS, Kwon T, Kim S (2014). IL-32theta downregulates CCL5 expression through its interaction with PKCdelta and STAT3. Cell Signal.

[45] Kim MS, Kang JW, Jeon JS, Kim JK, Kim JW, Hong J (2015). IL-32theta gene expression in acute myeloid leukemia suppresses TNF-alpha production. Oncotarget.

[46] Kim MS, Kang JW, Lee DH, Bak Y, Park YS, Song YS (2014). IL-32theta negatively regulates IL-1beta production through its interaction with PKCdelta and the inhibition of PU.1 phosphorylation. FEBS Lett.

[47] Choi J, Bae S, Hong J, Ryoo S, Jhun H, Hong K (2010). Paradoxical effects of constitutive human IL-32{gamma} in transgenic mice during experimental colitis. Proc Natl Acad Sci U S A.

[48] Choi JD, Bae SY, Hong JW, Azam T, Dinarello CA, Her E (2009). Identification of the most active interleukin-32 isoform. Immunology.

[49] Wong CK, Dong J, Lam CW (2014). Molecular mechanisms regulating the synergism between IL-32gamma and NOD for the activation of eosinophils. J Leukoc Biol.

[50] Bang BR, Kwon HS, Kim SH, Yoon SY, Choi JD, Hong GH (2014). Interleukin-32gamma suppresses allergic airway inflammation in mouse models of asthma. Am J Respir Cell Mol Biol.

[51] Busse WW, Lemanske RF (2001). Asthma. N Engl J Med.

[52] Bateman ED, Hurd SS, Barnes PJ, Bousquet J, Drazen JM, FitzGerald M (2008). Global strategy for asthma management and prevention: GINA executive summary. Eur Respir J.

[53] Kay AB (2006). The role of T lymphocytes in asthma. Chem Immunol Allergy.

[54] Barnes PJ (2008). Immunology of asthma and chronic obstructive pulmonary disease. Nat Rev Immunol.

[55] Simpson JL, Scott R, Boyle MJ, Gibson PG (2006). Inflammatory subtypes in asthma: assessment and identification using induced sputum. Respirology.

[56] Haldar P, Brightling CE, Hargadon B, Gupta S, Monteiro W, Sousa A (2009). Mepolizumab and exacerbations of refractory eosinophilic asthma. N Engl J Med.

[57] Woodruff PG, Modrek B, Choy DF, Jia G, Abbas AR, Ellwanger A (2009). T-helper type 2-driven inflammation defines major subphenotypes of asthma. Am J Respir Crit Care Med.

[58] Mortaz E, Masjedi MR, Allameh A, Adcock IM (2012). Inflammasome signaling in pathogenesis of lung diseases. Curr Pharm Des.

[59] dos Santos G, Kutuzov MA, Ridge KM (2012). The inflammasome in lung diseases. Am J Physiol Lung Cell Mol Physiol.

[60] Kwon JW, Chang HS, Heo JS, Bae DJ, Lee JU, Jung CA (2017). Characteristics of asthmatics with detectable IL-32gamma in induced sputum. Respir Med.

[61] Meyer N, Christoph J, Makrinioti H, Indermitte P, Rhyner C, Soyka M (2012). Inhibition of angiogenesis by IL-32: possible role in asthma. J Allergy Clin Immunol.

[62] Gasiuniene E, Lavinskiene S, Sakalauskas R, Sitkauskiene B (2016). Levels of IL-32 in serum, induced sputum supernatant, and bronchial lavage fluid of patients with chronic obstructive pulmonary disease. COPD.

[63] Wang Y, Zhou B, Zhao Y, Yu X, Liu Y, Zhang L (2016). Association of Plasma IL-32 levels and gene polymorphisms with systemic lupus Erythematosus in Chinese Han population. Dis Markers.

[64] Xuan W, Huang W, Wang R, Chen C, Chen Y, Wang Y (2017). Elevated circulating IL-32 presents a poor prognostic outcome in patients with heart failure after myocardial infarction. Int J Cardiol.

[65] Ha YJ, Park JS, Kang MI, Lee SK, Park YB, Lee SW. Increased serum interleukin-32 levels in patients with Behcet's disease. Int J Rheum Dis. 2017. [Epub ahead of print]. 10.1111/1756-185X.13072 28378461

[66] Mun SH, Kim JW, Nah SS, Ko NY, Lee JH, Kim JD (2009). Tumor necrosis factor alpha-induced interleukin-32 is positively regulated via the Syk/protein kinase Cdelta/JNK pathway in rheumatoid synovial fibroblasts. Arthritis Rheum.

[67] Lin X, Yang L, Wang G, Zi F, Yan H, Guo X (2017). Interleukin-32alpha promotes the proliferation of multiple myeloma cells by inducing production of IL-6 in bone marrow stromal cells. Oncotarget.

